# P40 expression in small cell lung cancer: The presence of p40‐positive cells does not always indicate squamous differentiation

**DOI:** 10.1111/1759-7714.13062

**Published:** 2019-04-07

**Authors:** Naoki Nakajima, Akihiko Yoshizawa, Koki Moriyoshi, Makoto Sonobe, Toshi Menju, Shinji Sumiyoshi, Hiroshi Date, Hironori Haga

**Affiliations:** ^1^ Department of Diagnostic Pathology Kyoto University Hospital Kyoto Japan; ^2^ Department of Diagnostic Pathology National Hospital Organization Kyoto Medical Center Kyoto Japan; ^3^ Department of Thoracic Surgery Kyoto University Hospital Kyoto Japan

**Keywords:** Diagnosis, immunohistochemistry, small cell lung cancer, TP63 protein

## Abstract

**Background:**

Small cell lung cancer (SCLC) is normally diagnosed with hematoxylin and eosin stains, although some cases require immunohistochemistry (IHC). P40 is highly sensitive and specific for squamous cell carcinoma and is thus considered the best marker for this cancer. However, the status of p40 expression in SCLC is not well known. The aim of this study was to analyze p40 expression in resected SCLC using IHC.

**Methods:**

Forty‐four surgically resected SCLC cases were enrolled. Clinical data were obtained from the patients’ medical records. Pathologists blinded to the patients’ clinical data reviewed the SCLC slides. IHC was performed on a representative slide of each case.

**Results:**

Although p40 was not diffusely expressed in any of the SCLC cases, p40‐positive cells were observed in the tumors in 15 cases (34.1%). Ten of these exhibited p40 in < 1% of tumor cells. In the remaining five cases, 1–5% of tumor cells expressed p40, and in three of these, the cells expressing p40 also expressed TTF‐1 and neuroendocrine markers. There was no statistically significant relationship between p40 positivity and any other clinicopathological characteristics.

**Conclusions:**

Some resected SCLCs express p40 focally. This result suggests that the presence of positive p40 cells does not exclude a diagnosis of SCLC. Thus, small biopsy or cytology specimens with p40‐positive cells must be diagnosed carefully.

## Introduction

Small cell lung cancer (SCLC) accounts for approximately 14% of all newly diagnosed lung cancer cases.^1^ SCLCs are normally diagnosed with hematoxylin and eosin (HE) staining using small biopsy and cytology specimens.^2^ However, some cases require immunohistochemistry (IHC) to differentiate them from lymphomas, low‐grade neuroendocrine tumors, or basaloid squamous cell carcinomas (SCCs).

Several SCC markers can be detected using IHC: CK5/6, DSM3, p40, and p63. P40, an isoform of p63 (deltaNp63), is a highly sensitive and specific marker of SCC. In previous reports, both the sensitivity and specificity of p40 for diagnosing pulmonary SCC have been almost 100%.^3^, ^4^, ^5^ Thus, p40 is known as the best marker of this cancer.^2^ However, the status of p40 expression in SCLC is not well known.^6^, ^7^, ^8^ The aim of this study was to analyze p40 expression in resected SCLC using IHC.

## Methods

### Patient cohort

We prepared two patient datasets for this study. First, between January 2005 and December 2017, 2486 patients with primary lung carcinoma underwent pulmonary resection at Kyoto University Hospital. Thirty‐eight of these cases (1.5%) were SCLC. One patient administered chemotherapy before surgery and four patients whose clinical data were unavailable were excluded. The remaining 33 cases were enrolled. Second, between January 2009 and December 2017, 625 patients with primary lung carcinoma underwent pulmonary resection at the National Hospital Organization, Kyoto Medical Center. Eleven of these 625 cases (1.8%) were SCLC. All 11 cases were enrolled in this study. Clinical data, including gender, age, smoking status, and serum tumor marker levels of progastrin releasing peptide (pro‐GRP) and neuron‐specific enolase (NSE), were obtained from the medical records of both patient datasets.

Patients were informed about the possibility of their tissue samples being used for academic research, and were advised that they could opt out of this research. The institutional ethics committees of Kyoto University Hospital (R1324‐1) and the National Hospital Organization Kyoto Medical Center (18–041) granted approval of this retrospective study.

### Histologic evaluation

All resected specimens were fixed in formalin, sectioned, and stained with HE in the conventional manner. Elastic stains (Elastica van Gieson, Elastica‐Masson, or Victoria Blue, Muto Pure Chemicals, Tokyo, Japan) were also performed to detect invasion of the pleura or vessels. Two pathologists blinded to the patient's clinical data reviewed the SCLC slides. All cases were classified according to 2015 World Health Organization criteria.^1^ SCLC was classified based on specific histological and cytological features on HE staining; the decision was not altered on the basis of IHC findings. In addition, tumor staging was performed according to the 8th edition of the International Union Against Cancer Tumor Node Metastasis Classification.^9^ Lymphatic, vascular, and pleural invasion, and/or spread through air spaces, were assessed.

### Immunohistochemistry

IHC was performed on a representative slide of each case. We used a specific antibody to p40 (clone BC28; Roche, Basel, Switzerland). The association between p40 and protein expression was evaluated using the following antibodies: TTF‐1 (clone 8G7G3/1), CgA (polyclonal A0430) and Ki‐67 (clone MIB‐1, Dako, Glostrup, Denmark); SYN (clone MRQ‐40) and CK5 (clone SP27, Roche); CD56 (clone 1B6, Leica Microsystems, Wetzlar, Germany); and DSC3 (clone Dsc3‐U114, PROGEN Biotechnik, Heidelberg, Germany). IHC was performed with an automatic immunostainer (Benchmark, Ventana Medical Systems, Tucson, AZ, USA) according to the manufacturer's instructions. For CgA, SYN, and CD56, positive staining was defined as ≥ 10% positive tumor cells according to 2015 World Health Organization classification criteria for large cell neuroendocrine carcinoma.^1^ For TTF‐1 and DSM3, positive staining was also defined as ≥ 10% positive tumor cells. Co‐expression of p40 with other antigens was determined by comparing two slides.

### Statistical analysis

Data were analyzed using JMP Pro version 12.2.0. Comparisons between two groups were performed using Fisher's exact test to analyze categorical variables. Statistical significance was indicated at *P* < 0.05.

## Results

**Figure 1:**
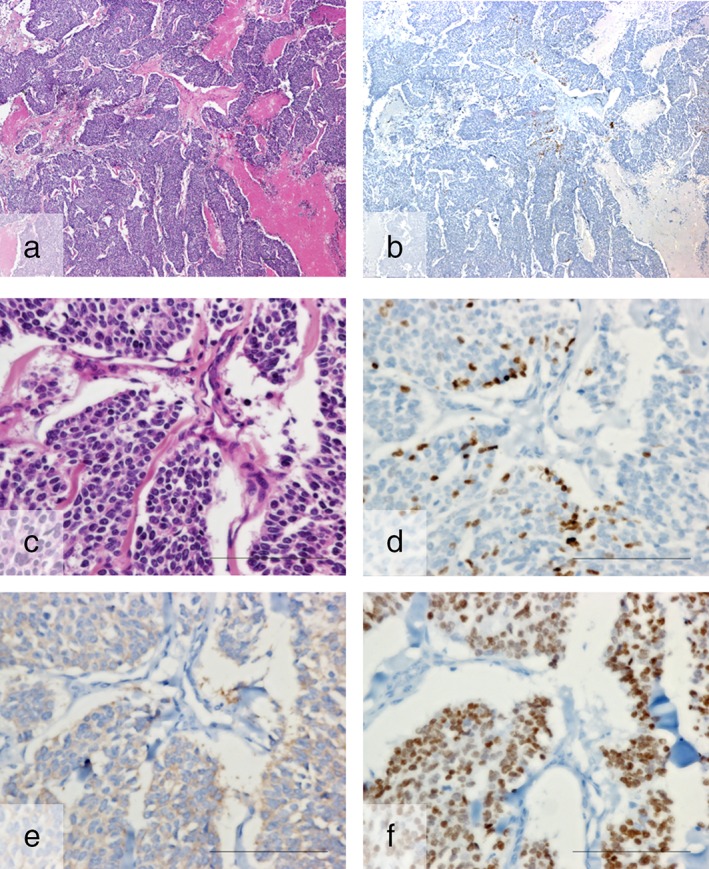
A representative case of p40‐positive small cell lung cancer. (**a**,**c**) Hematoxylin and eosin staining. Immunohistochemical images of (**b**,**d**) p40, (**e**) SYN, and (**f**) TTF‐1. (**b**) Low power view shows foci expressing p40. (**c**,**d**) High power views show that the morphology of p40‐expressing cells is the same as p40‐negative cells. (**e**,**f**) Co‐expression of SYN and TTF‐1 is observed. Bars indicate 100 μm.

Of the 44 total cases studied, 30 were pure SCLC, four were combined SCLC and SCC, nine were combined SCLC and adenocarcinoma, and one was combined SCLC and large cell neuroendocrine tumor. The clinicopathological features of these 44 cases are listed in Tables 1 and 2. Although p40 was not diffusely expressed in any of the SCLC cases, p40‐positive cells were observed in 15 cases (34.1%): in 10 cases, < 1% of tumor cells were positive in a whole slide; in three cases, 1–5% of tumor cells were positive in a whole slide; and in two cases, > 10% of tumor cells were positive in a focal area (Fig 1a–d). The cytological features of the p40‐positive tumor cells did not differ from those of surrounding p40‐negative cells (Fig 1c,d); the p40‐positive tumor cells were morphologically diagnosed as SCLC. Regarding p40 positivity of the corresponding biopsy specimens, biopsy before resection was performed in only seven cases. Among these seven cases, one biopsy specimen contained tumor tissue and no p40‐positive cells were observed.

**Table 1:** Association between p40 expression and clinical characteristics of small cell lung cancer

Characteristics	Expression of p40		*P*
	Positive (≥ 1%)	Negative (< 1%)	
Total	5	39	
Age			
≥ 65	3	31	0.59
< 65	2	8	
Gender			
Male	2	29	0.32
Female	3	10	
Smoking			
Never	0	2	1.00
Ever	5	37	
T Stage			
T1a	0	3	0.87
T1b	2	6	
T1c	1	5	
T2a	1	17	
T2b	0	2	
T3	1	5	
T4	0	1	
N stage			
N0	2	26	0.62
N1‐3	3	8	
M Stage			
M0	5	38	1.00
M1	0	1	
Stage			
IA1	0	3	0.87
IA2	1	3	
IA3	1	2	
IB	1	12	
IIA	0	2	
IIB	1	8	
IIIA	1	4	
IIIB	0	0	
IV	0	1	

**Table 2:** Association between p40 expression and histological characteristics of SCLC

Characteristics	Expression of p40		*P*
	Positive (≥ 1%)	Negative (< 1%)	
Histological subtype			
SCLC	4	26	0.55
SCLC + SCC	1	3	
SCLC + ADC	0	9	
SCLC + LCNEC	0	1	
Pleural invasion			
Present	2	18	1.00
Absent	3	21	
Vascular invasion			
Present	4	24	0.64
Absent	1	15	
Lymphatic invasion			
Present	3	17	0.65
Absent	2	22	
STAS			
Present	4	29	1.00
Absent	1	10	

ADC, adenocarcinoma; LCNEC, large cell neuroendocrine tumor; SCC, squamous cell carcinoma; SCLC, small cell lung cancer; STAS, spread though air spaces.

We examined five cases in which > 1% of the tumor cells expressed p40 to determine whether p40‐positive tumor cells also expressed TTF‐1, neuroendocrine makers, or other SCC markers (Table 3). In a case of combined SCLC and SCC (case 2), IHC evaluation was performed on the SCLC area. In two of these cases (cases 1 and 3), p40‐positive tumor cells expressed TTF‐1, SYN, CD56, and CK5. In one case (case 5), p40‐positive tumor cells expressed TTF‐1, SYN, and CK5. In the remaining two cases (cases 2 and 4), p40‐positive tumor cells did not express TTF‐1, neuroendocrine markers, or CK5.

**Table 3:** Co‐expression of p40 and other antigens

Case	Histological diagnosis	TTF‐1	CD56	CgA	CK5	DSM3	SYN	Ki‐67 index†
1	SCLC	Present	Present	Absent	Present	Absent	Present	49.6
2‡	SCLC‐SCC	Absent	Absent	Absent	Absent	NA	Absent	NA
3	SCLC	Present	Present	Absent	Present	Absent	Present	72.2
4	SCLC	Absent	Absent	Absent	Absent	NA	Absent	88.8
5	SCLC	Present	NA	Absent	Present	Absent	Present	62.2

† Ki‐67 index of the total tumor.

‡ Evaluation of immunohistochemistry in this case of combined small cell lung cancer (SCLC) and squamous cell carcinoma (SCC) was performed on the SCLC area. NA, not available.

Information about the serum pro‐GRP level was available in 30 cases. The mean serum pro‐GRP level was 78.2 pg./mL (range: 20.7–317 pg./mL) in cases with p40‐negative tumors and 78.8 pg./mL (range: 27.9–143 pg./mL) in cases with p40‐positive tumors. Information about the serum NSE level was available in 32 cases. The mean serum NSE level was 12.57 ng/mL (range: 4.10–26.97 ng/mL) in cases with p40‐negative tumors and 15.63 ng/mL (range: 11.05–21.99 ng/mL) in cases with p40‐positive tumors.

There was no statistically significant relationship between p40 positivity and any other clinicopathological characteristics (Tables 1, 2).

## Discussion

P40 is regarded as the best marker of SCC; however, the status of p40 expression in SCLC has not been well studied. We showed that 34.1% of resected SCLCs express p40 focally. These results indicate that p40 positivity does not always exclude a diagnosis of SCLC.

Few reports mention p40 expression status in SCLC and most of these reported that p40 was not expressed in SCLC tumor cells.^6^, ^7^, ^10^ Brown *et al.* used a dual‐antibody cocktail to assess p40/TTF‐1, and positive nuclear red staining in > 1% of tumor cells was scored as positive for p40, showing that all SCLC cases (*n* = 35) were identified by strong positive staining for TTF‐1 and negative staining for p40.^7^ This result might indicate no or ≥ 1% expression of p40 in their series; however, we believe that it would have been difficult to recognize p40 positive cells because they used a dual‐antibody cocktail. Thus their result does not conflict with ours. Among 78 biopsies and 18 surgical specimens, Miyauchi *et al.* found no SCLC samples positive for p40; however, they may have ignored a small number of p40‐positive cells in SCLC because they did not report the threshold.^10^ Butonor *et al.* also did not observe nuclear p40 immunoreactivity in 27 biopsy samples or seven resection/autopsy specimens.^6^ In our study, the p40‐positive area in SCLC was very limited, thus we suggest that examination of a resected specimen is essential to determine whether SCLC expresses p40. The number of resected specimens in the study by Butonor *et al.* was smaller than ours, thus, our findings are more robust.

A study by Lilo *et al.* demonstrated that p40 was focally and weakly positive in 12.5% (1/8) of SCLC cases using fine needle aspiration material.^8^ This report supports our assumption that SCLC can express p40 focally. Tumor cells taken from a lung nodule with p40 expression may lead pathologists to diagnose the tumor as SCC; however, our study showed that p40 positivity does not always exclude SCLC.

Interestingly, we found co‐expression of p40 and TTF‐1 in our sample. Tanaka *et al.* reported that co‐expression of p40 and TTF‐1 was observed in a type of peripheral lung epithelial stem cell.^11^ This finding may indicate that co‐expression of p40 and TTF‐1 in SCLC implies stem cell‐like features rather than squamous cell differentiation.

There are some limitations to this study. First, our results were obtained using a small number of cases because patients with SCLC are usually treated with chemotherapy and/or radiotherapy and were thus excluded. However, to the best of our knowledge, our SCLC cohort contains the largest number of resected specimens used to estimate p40 expression among reported SCLC cohorts. Second, we could not perform immunofluorescence or double staining to confirm the co‐expression of p40 and other antigens because p40 was expressed focally. As an alternative, we compared two slides (e.g. p40 and CgA) to determine the presence of co‐expression. Third, most cases enrolled in this study were early stage SCLC, although most cases of SCLC are detected in advanced stage.^2^ The frequency of p40 positivity in advanced stage SCLC might be different from that in early stage SCLC. However, we consider it impossible to evaluate p40 status in advanced stage tumors.

In conclusion, we show that resected tissue from cases of SCLC express p40 focally. This result suggests that positivity for p40 does not exclude SCLC. Thus, small biopsy or cytology specimens with p40‐positive cells must be diagnosed carefully.

## Disclosure

No authors report any conflict of interest.
